# Cognitive and Emotional Appraisal of Motivational Interviewing Statements: An Event-Related Potential Study

**DOI:** 10.3389/fnhum.2021.727175

**Published:** 2021-09-22

**Authors:** Karen Y. L. Hui, Clive H. Y. Wong, Andrew M. H. Siu, Tatia M. C. Lee, Chetwyn C. H. Chan

**Affiliations:** ^1^Department of Rehabilitation Sciences, The Hong Kong Polytechnic University, Hong Kong, SAR China; ^2^The Hong Kong Society for Rehabilitation, Hong Kong, SAR China; ^3^State Key Laboratory of Brain and Cognitive Sciences, The University of Hong Kong, Hong Kong, Hong Kong, SAR China; ^4^Laboratory of Neuropsychology and Human Neuroscience, The University of Hong Kong, Hong Kong, Hong Kong, SAR China; ^5^Department of Health Sciences, Brunel University, London, United Kingdom; ^6^Department of Psychology, The Education University of Hong Kong, Hong Kong, SAR China

**Keywords:** motivational interviewing, linguistics, readiness, return to work, ERP, P200, N400, LPC

## Abstract

The counseling process involves attention, emotional perception, cognitive appraisal, and decision-making. This study aimed to investigate cognitive appraisal and the associated emotional processes when reading short therapists' statements of motivational interviewing (MI). Thirty participants with work injuries were classified into the pre-contemplation (PC, *n* = 15) or readiness stage of the change group (RD, *n* = 15). The participants viewed MI congruent (MI-C), MI incongruent (MI-INC), or control phrases during which their electroencephalograms were captured. The results indicated significant Group × Condition effects in the frontally oriented late positive complex (P600/LPC). The P600/LPC's amplitudes were more positive-going in the PC than in the RD group for the MI congruent statements. Within the PC group, the amplitudes of the N400 were significantly correlated (*r* = 0.607–0.649) with the participants' level of negative affect. Our findings suggest that the brief contents of MI statements alone can elicit late cognitive and emotional appraisal processes beyond semantic processing.

## Introduction

The goal of counseling is to help clients learn new ways of being and to develop new memories and connections, ultimately facilitating the change to desirable behavior (Ivey et al., 2017). There are four different components in counseling, namely: attention, emotion perception, cognitive evaluation, and decision-making. Attention is regarded as a prerequisite, enabling the client to focus on interacting with the therapist (Field et al., 2015) and is also essential in driving the effect of the counseling process, enhancing learning, memory, and change (Ivey et al., 2017). Emotion perception and cognitive evaluation are separate processes but may be sequential or concurrent (Hundrieser and Stahl, 2016). Emotion perception is bottom-up when evoked by incoming visual or auditory stimuli (Ivey et al., 2017) or by information or events relating to previous experiences (Field et al., 2015, 2019). Cognitive evaluation commonly occurs when clients form an “insight” into the beliefs or views of self, others, and the world (Miller and Taylor, 2016), or form a “thought” of change in dysfunctional beliefs (Bruijniks et al., 2018). Decision-making is the process before the actual change takes place in counseling. Clients may need to access their long-term memory and make conscious decisions to feel, think, and act differently (Miller and Taylor, 2016).

Most neuroscience-related studies on counseling are based on functional brain imaging, and only a few studies have used event-related potential (ERP). The event-related potential has a good temporal resolution, which is useful for disentangling the complex neural processes associated with counseling processes. However, all the ERP studies reviewed aimed to assess pre-post treatment outcomes such as whether intervention can normalize hyperactive ERPs due to psychological disorders or to predict the level of treatment effectiveness (e.g., Ladouceur et al., 2018; Hajcak et al., 2019). The findings of these studies do not seem to have improved understanding of the mechanisms underlying the counseling processes. This study aims to understand the potential processes related to the way individuals perceive and respond to verbal contents in counseling. Going beyond the outcome of the study, we have designed a linguistic paradigm and employed an event-related potential method for elucidating these processes.

Motivational interviewing (MI) is a goal-oriented counseling technique designed primarily to promote health behavior change (Miller and Rollnick, 2013). The two primary MI features found to contribute to behavioral change are the change agent's (i.e., therapist) empathy and the verbal contents in the interaction with a client (Miller and Rose, 2009; Copeland et al., 2015). The neural processes associated with the change talks are inhibiting reward-related processes (Feldstein Ewing and Houck, 2015), arousing self-awareness and introspection (Feldstein Ewing et al., 2014). This study is the first in MI to focus on the neural processes associated with participants' exposure to MI change talk contents. We employed a customized linguistic task and ERP to disentangle the possible perception and evaluation processes associated with the contents of the MI statements.

Previous studies reported distinctive psychophysiological markers reflecting both early and late neural processes related to linguistic stimuli in decision making. The early neural process is the instant perceptual response to the incoming stimuli reflects by the frontal P200. Sarlo et al. (2012) used contrasted scenarios which aroused moral dilemmas among the participants. More positive-going frontally oriented P200 was found to relate to the instant affective response to the presenting stimuli. The amplitudes of P200 were found to be modulated by the positive vs. negative-context adjectives (Li et al., 2016) and emotion-loaded stimuli (Carretié et al., 2001; Huang and Luo, 2006). P200 was also associated with enhanced attention to stimuli with emotional valence (Proverbio et al., 2020). Another ERP related to linguistic stimuli is the frontally oriented N400, which reflects the evaluation of the stimulus contents (Kutas and Federmeier, 2000). In moral decision making, the evaluation was found to compare with an individual's prior knowledge and experience. Hundrieser and Stahl (2016) reported that the value-inconsistent trials elicited more negative-going N400 than the value-consistent trials. Another study suggested that N400 was related to the cognitive evaluation of semantic information (Peng et al., 2020). The evaluation process was further explained to involve cognitive integration of the incoming semantics with those stored in the lexical long-term memory (Bechtold et al., 2019). The third ERP of interest is the extended centro-frontal P300 forming the P600 or late positive component (LPC). The P600 and LPC will be used interchangeably. The LPC reflected emotional appraisal of semantic contents (Delogu et al., 2019). More positive-going LPC was associated with emotionally-loaded words or pictures in contrast to neutral stimuli (Hajcak et al., 2010). In moral decision making, increased in the LPC amplitudes were found in stimuli containing dilemma conflicts and negative emotions (Zhan et al., 2018). It was postulated that the more positive LPC would represent the emotional appraisal process in conflict resolution (McKay et al., 2017). Taken together, the psychophysiological markers adopted in this study are the P200 and LPC which reflects emotional perception and evaluation processes, while the N400 would reflect cognitive appraisal of the semantic MI contents.

Participants in this study were adults who had been injured at work and were receiving a return-to-work rehabilitation service. They were further classified into two groups according to Prochaska et al.'s (1985) readiness for change model: contemplation to action groups (RD, higher readiness) and pre-contemplation groups (PC, lower readiness). We hypothesized that the PC group would have a more positive-going P200 and a more negative-going N400 than those in the RD when reading the MI-congruent statements. It was also hypothesized that the PC group would show a more positive-going LPC than the RD group, suggesting the former group would find the MI-congruent content more consistent in their evaluation than the latter group.

## Materials and Methods

### Participants

We recruited forty adults affected by work injuries and receiving return-to-work rehabilitation. They were service users of hospital out-patient clinics or community rehabilitation centers. The inclusion criteria were: (1) an age range of 18–60; (2) a secondary school education or above; (3) a history of work-related injuries within the last 3 years; (4) either in the pre-contemplation (PC) or contemplation to action stages of readiness of return-to-work (RD) as defined by the Chinese version of Lam assessment of employment readiness (C-LASER) (Chan et al., 2006). The exclusion criteria were: (1) illiterate or unable to read traditional Chinese characters; (2) reported a diagnosis of other significant medical or psychiatric illness. The participants were informed of the purposes of the study and gave voluntary consent. Each participant received HK$500 for covering the transportation costs. Ethics approval was granted from the university's ethics committee, where the study was carried out.

Among the total sample (*N* = 40), five from each group were excluded after the data collection due to excessive artifacts or alpha waves found in the data. The final sample size entered into the data analyses was 15 in each of the PC and RD groups. For these 30 participants, 11 (36.7%) were male and 19 (63.3%) were female, with a mean age of 46.8 (SD: 7.7). Nineteen of them (63.3%) had a secondary school education and 11 (36.7%) had a University education or above. For the PC group, 7 (46.7%) were male and 8 (53.3%) were female, with a mean age of 47.5 (SD: 6.4). For the RD group, 4 (26.7%) were male and 11 (73.3%) were female, with a mean age of 46.1 (SD: 8.9). No significant between-group differences were found in the age, gender composition, and education level (Table 1).

**Table 1:** Demographic characteristics and test results of participants.

		**Overall (*N* = 30)**	**PC group (*n* = 15)**	**RD group (*n* = 15)**	***X^**2**^***	***P-value***
Age (years)		46.80 (7.7)	47.5 (6.4)	46.1 (8.9)	0.52^a^	0.61
Gender	Male	11 (36.7%)	7 (46.7%)	4 (26.7%)	1.29^b^	0.26
	Female	19 (63.3%)	8 (53.3%)	11 (73.3%)		
Education Level	Secondary	19 (63.3%)	9 (60.0%)	10 (66.7%)	0.14^b^	0.71
	Tertiary	11 (36.7%)	6 (40.0%)	5 (33.3%)		
C-LASER	Pre-contemplation	15.07 (3.9)	17.9 (2.6)	11.7 (2.0)	6.93^a^	<0.001^**^
	Contemplation	13.25 (3.3)	11.9 (2.9)	14.9 (3.1)	−2.66^a^	0.013
	Preparation	13.07 (3.1)	11.9 (3.1)	14.5 (2.4)	−2.45^a^	0.02
	Action	12.21 (3.7)	10.3 (3.2)	13.9 (3.6)	−2.49^a^	0.02
STAI-C (State)		54.1 (9.3)	53.0 (11.1)	55.13 (7.4)	−0.6^a^	0.54
PANAS	Positive	24.8 (7.9)	27.67 (7.3)	22.0 (7.6)	2.08^a^	0.05
	Negative	18.3 (6.0)	20.1 (6.5)	16.6 (5.1)	1.62^a^	0.12

a *T-values*.

b *Chi-square*.

Bonferroni adjustment for significance level of C-LASER is 0.05/4 = 0.013 and PANAS is 0.05/2 = 0.025. The significance level of Pre-contemplation is smaller than 0.01/4 after correction and is indicated with **.

### Study Design

Participants in this study were injured workers classified into two readiness groups, i.e., PC and RD. The work disability prevention model stipulated that injured workers would make the return to work decisions based on the person, workplace, compensation, and health care system (Nowrouzi-Kia et al., 2021). Injured workers were revealed to have higher risks in developing psychological issues such as anxiety, depression, and posttraumatic stress disorder (Giummarra et al., 2018). Because of these unique circumstances faced by the injured workers, this study did not adopt a non-injured worker control group. Instead, the testing of the hypotheses was between the two injured worker groups. Each participant was to complete three instruments that measure change, mental state, and general health. The participant then received a short mindfulness training before completing an experimental task.

### Experimental Task

#### Construction MI Statements

There were three types of statements: MI-congruent, MI-incongruent, and a control. The statement stimuli were made up of: (1) seven Chinese characters; (2) the same syntactic structure; (3) the last three characters critical to the behavioral responses (three-critical-characters; Li et al., 2016). There are three types of 120 statements. The MI-congruent statements (MI-C) convey positive values consistent with the MI approach. The MI-incongruent statements (MI-INC) convey negative values unsupportive of return-to-work. The control statements (MI-CON) had the three-critical-characters of fruit names. Examples of the statements are: MI-C [尋找工作好重要 (Seeking job is important)], MI-INC [尋找工作很困難 (Seeking job is difficult)], and MI-CON 富士蘋果有四個 (There is four Fuji apple)]. The noun vs. verb syntax of the statements is comparable among the three conditions (see Supplementary Material). The nouns and verbs of these statements are identified below:

Noun Noun Verb

MI-C: 尋找(Seeking) |工作(job) |好重要(is important)

MI-INC: 尋找(Seeking) |工作(job) |很困難(is difficult)

MI-CON: 富士(Fuij) |蘋果(apple) |有四個(There is four).

#### The Task

**Figure 1:**
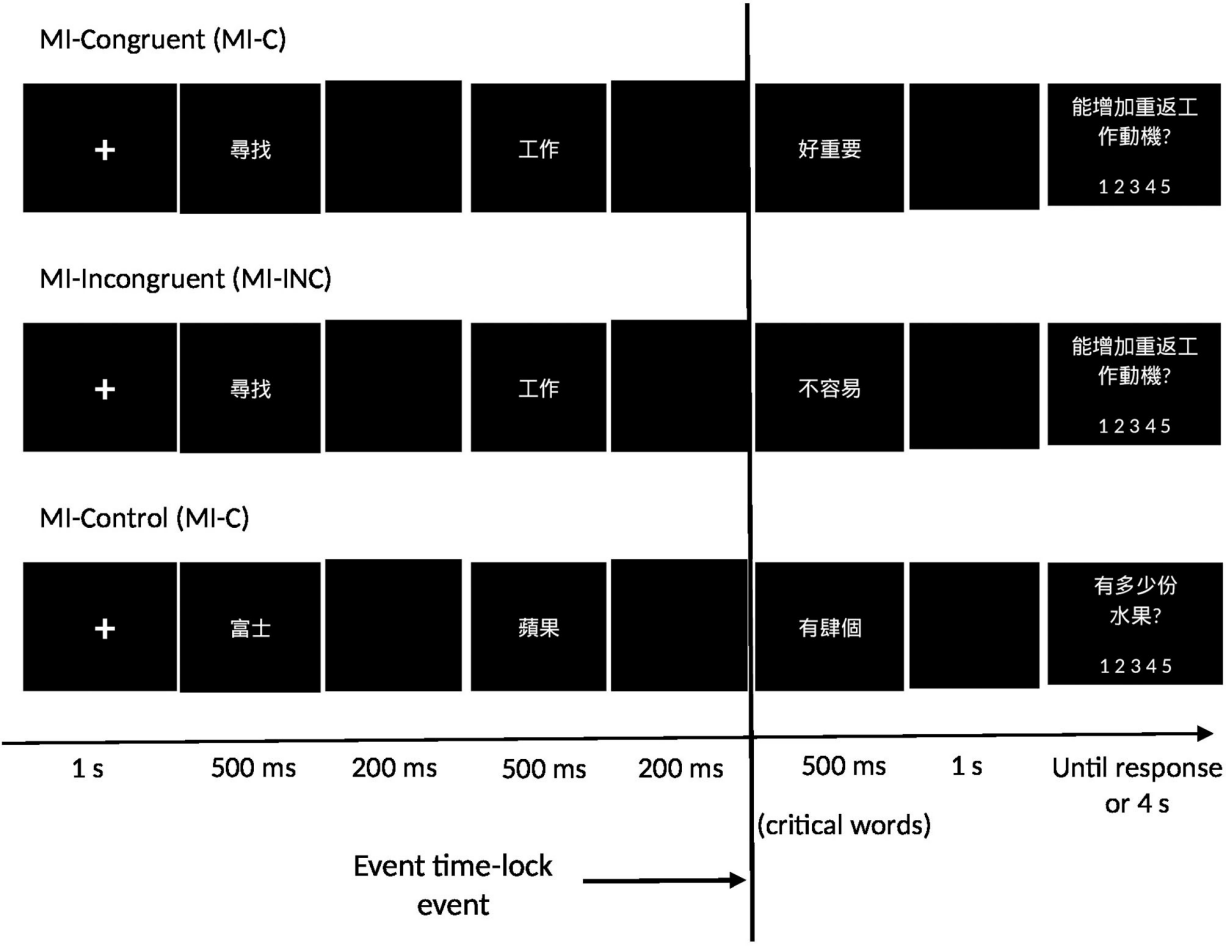
Design of the trials in the Ml-congruent, Ml-incongruent, and Ml-control conditions.

The delivery of the statements and the type of response adopted the rapid serial visual presentation (RSVP) method commonly used in psycholinguistic studies (Chen et al., 2013; Hundrieser and Stahl, 2016). The design of the statement trial is presented in Figure 1. The participants read an MI-C statement presented on the computer screen in the form of three consecutive texts and then gave a response to the question: “Would it enhance your motivation for return to work?” (for RTW readiness) by pressing on the 1–5 keys on the number pad. The same question was repeated in all the MI-C and MI-INC statements. The responses to the MI-CON statements were to indicate the number of fruits conveyed in the text. After completing the task, the participants rated the statements according to their relevance to their return-to-work situation on a 5-point rating scale. The 360 statement trials were organized in random order into 24 blocks. The composition and presentation of the task used STIM2 software (Compumedics Neuroscan, USA). The duration of the task was around 4.5 min.

#### Procedures

The participant provided personal information and completed there clinical instruments. They are the Chinese version Lam Assessment of Employment Readiness (C-LASER; Lam et al., 2010), the Positive and Negative Affect Schedule (PANAS; Watson et al., 1988), and the state-anxiety subscale of the Chinese version of the State-Trait Anxiety Inventory (C-STAI; Shek, 1993).

**Figure 2:**
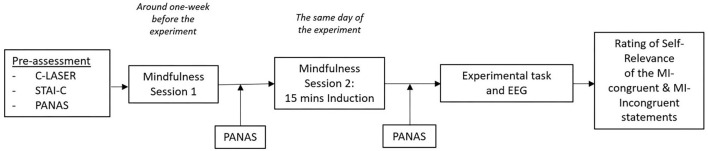
The flow of the experiment.

The flow of the experiment is presented in Figure 2. Before engaging in the experimental task, the participant completed 15 min of mindfulness training provided by the first author. The purpose of the training was to minimize excessive emotional responses to the statements throughout the experiment. Previous studies have reported the 15-min mindfulness induction session as an effective emotional regulation strategy (Eddy et al., 2015). Participants completed PANAS for the second time after the training session. The RD group showed significant decreases in the PANAS negative affect scores (*t* = −3.966, *p* = 0.001), which was not the case for the PC group (Table 1). Both groups did not show significant changes in the positive affect PANAS scores.

The EEG experiment was carried out in a soundproof chamber. The participant sat at a table facing a 15-inch computer monitor at a distance of 60 cm. Each participant completed 20 statement practice trials. All the texts were at the center of the screen, and were white against a dark background and in *KaiTi* 24-point size font. Standardized instructions were given to the participant on the task procedure, such as minimizing eye blinks and keeping eye fixation at the center of the screen.

#### EEG Acquisition and Pre-processing

During the task, electrical signals were recorded with a 32-channel cap using NuAmps Digital EEG Amplifier and CURRY 7 (Compumedics Neuroscan, USA). The reference electrode was mounted on the right mastoid, and the ground electrode was placed on the forehead. Impedances were maintained at 5 kΩ or below. Pre-processing was performed with EEGLAB (version 2019.1; Delorme and Makeig, 2004). Data were high pass filtered at 0.1 Hz. Independent Component Analysis (ICA) decomposition was performed using AMICA (Palmer et al., 2012). Noise components were detected using ICLabel (Pion-Tonachini et al., 2019) and then checked manually and removed from the data. The processed data were then epoched with a window of 200 ms prior to and 1500 ms following the target stimulus (i.e., three-critical-characters on the 5th screen) using ERPLab (version 8.0, Lopez-Calderon and Luck, 2014). Grand averages for each participant were calculated and exported. Participants who did not reach a minimum of 60 passed trials in each of the three conditions were also excluded from the analyses (Huffmeijer et al., 2014).

### Data Analysis

The reaction time (RT) and the return-to-work readiness (RTW-R) rating of participants' behavioral data were collected using STIM2. The content relevance ratings (C-REL) were collected using a questionnaire once the participants had completed the EEG task. Repeated measure ANOVAs were conducted on the RTs, and the RTW-R and C-REL to test the between-group (PC and RC) and between-condition (MI-C and MI-INC) differences. All analyses were performed using the IBM SPSS for Windows version 25.0.

The processed and validated data of the EEG were imported to the ERP-PCA toolbox (version 289; Dien, 2010). A Temporal Principal Component Analysis (PCA) decomposition was conducted with Promax rotation for identifying the P200, N400, and LPC components The amplitude of each identified component at the peak channel was extracted and analyzed with a linear mixed-effect model using R (v3.6.2), R-package lmer (v1.1-21), and lmerTest (v3.1-1). The Group (PC and RD), Condition (MI-C, MI-INC, and MI-CON), and their interactions were entered as fixed effects, while the participants were entered as random effects. For models with significant Group × Condition effects, *post-hoc* pairwise analyses were conducted on between-group differences for each condition and between-condition differences for each group. Pearson's correlation was used to explore the relationships between the identified ERPs' amplitudes and the behavioral and clinical assessment and test results. To control for a potential type I error, the significance level for the correlation coefficients was corrected with Bonferroni adjustment of *p* < 0.017 (three ERPs). To test whether gender composition would confound the results, the ANOVA models were rerun by replacing the independent variable Group with the Gender of the participants. We presumed that gender was not associated with the potentials of the ERP components. Additionally, we could incorporate Gender as an extra independent variable to statistically adjust for the effect of Gender. However, due to the small sample size and collinearity with the Group variable, we considered that statistical correction was not appropriate. Alternatively, in a supplementary analysis, we also tested if adding Gender as an independent variable into the model would improve the model evidence (Burnham and Anderson, 2002, Supplementary Table 1). The goodness of fit tests Akaike information criterion (AIC, Akaike, 1974) and Bayesian information criterion (BIC, Schwarz, 1978) were adopted for reflecting the influence of the gender effect on the models. Include both the AIC and BIC would increase the robustness of the results as they have different requirements on the number of predictors. We conjectured that incorporating gender into the models would not improve the model evidence. The goodness-of-fit tests were performed with the R/lmerTest.

## Results

### Behavioral

No significant between-group differences were found in the scores of the PANAS and C-STAI. For the C-LASER, as expected, the PC group had a significantly higher mean score in the “Pre-contemplation” subscale than the RD group while the RD group had a significantly higher score in the “Contemplation” subscale than the PC group (Table 1). The Condition effect on the reaction times was statistically significant, *F*(1, 28) = 33.66, *p* < 0.005 (Table 2). However, the Group, [*F*_(1, 28)_ = 0.11, *p* = 0.741], and Group × Condition effects, [*F*_(1, 28)_ = 0.16, *p* = 0.688], were not significant. The mean RT of the MI-C trials (Mean = 932.31 ± 72.59 ms) was significantly shorter than that of the MI-INC trials (Mean = 1115.53 ± 83.46 ms), *t* = −5.887, *p* < 0.005.

**Table 2:** Comparisons of the response times (RTs), return to work readiness (RTW-R) ratings, and content relevance (C-REL) ratings for the MI-congruent and incongruent statements between participants in the pre-contemplation (PC) and readiness (RD) groups.

	**RT (ms)**		**RTW-R ratings**		**C-REL ratings**	
	**Mean**	***SEM***	**Mean**	***SEM***	**Mean**	***SEM***
PC group						
MI-C	942.6	123.3	3.72	0.13	3.17	0.28
MI-INC	11148.2	142.1	2.91	0.10	2.95	0.14
RD group						
MI-C	904.1	81.2	3.99	0.16	3.67	0.24
MI-INC	1082.9	92.3	2.67	0.16	2.55	0.16

*SEM, standard error of the mean; RT, reaction time; RTW-R, return to work readiness ratings; C-REL, content relevance ratings*.

#### RTW Readiness Ratings

The Condition effect on the RTW-R ratings was statistically significant, [*F*_(1, 28)_ = 66.61, *p* < 0.005]. However, the Group [*F*_(1, 28)_ = 0.01, *p* = 0.922] and Group × Condition effects, [*F*_(1, 28)_ = 3.72, *p* = 0.64] were not statistically significant. The mean RTW-R rating for the MI-C trials (Mean = 3.86 ± 0.10) was significantly higher than that for the MI-INC trials (Mean = 2.79 ± 0.10), *t* = 7.804, *p* = *p* < 0.005.

#### Statement Content Relevance Rating

The Group × Condition effects on the C-REL were statistically significant, [*F*_(1, 28)_ = 5.38, *p* = 0.028]. The Condition effect was significant, [*F*_(1, 28)_ = 11.78, *p* = 0.002], but the Group effect was not significant, [*F*_(1, 28)_ = 0.05, *p* = 0.825]. In the RD group, the C-REL ratings of the MI-C trials were higher than those of the MI-INC trials, *t* = 3.81, *p* = 0.002, which was not the case for the PC group, *t* = 0.85, *p* = 0.41. No significant differences were found in the C-REL ratings of the MI-C statements between the two groups, *t* = −1.353 *p* = 0.187. The relationships between the C-REL ratings and RTW readiness ratings in the MI-C statements were moderate in the RD group, *r* = 0.658, *p* = 0.008 but not in the PC group, *r* = 0.416, *p* = 0.123.

### Event-Related Potential

**Figure 3:**
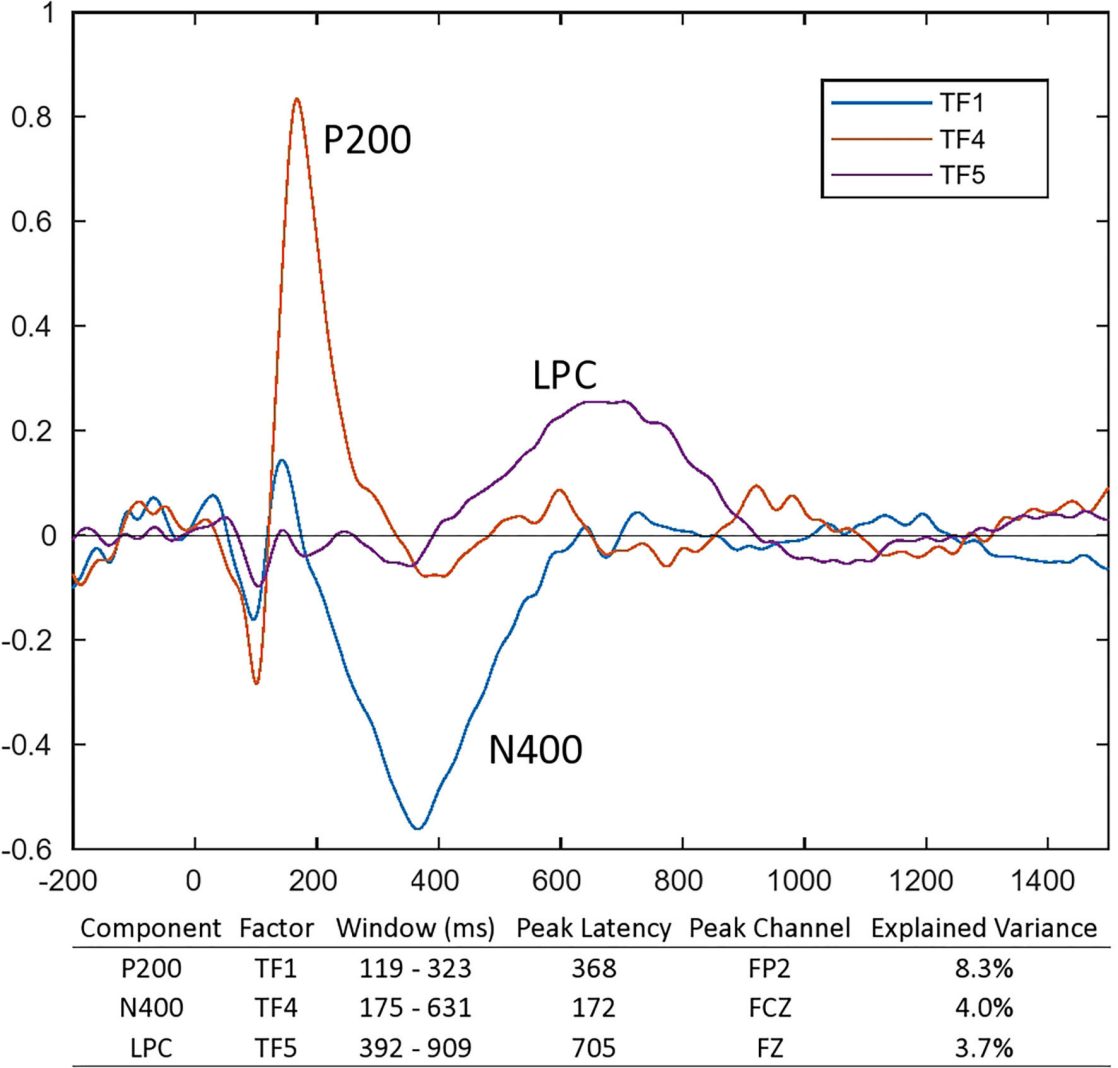
ERP waveforms and temporal factors identified based on the temporal principal component analysis. After temporal PCA, three out of the seven temporal components conformed to P200, N400, and LPC. TF denoted temporal factor.

**Figure 4:**
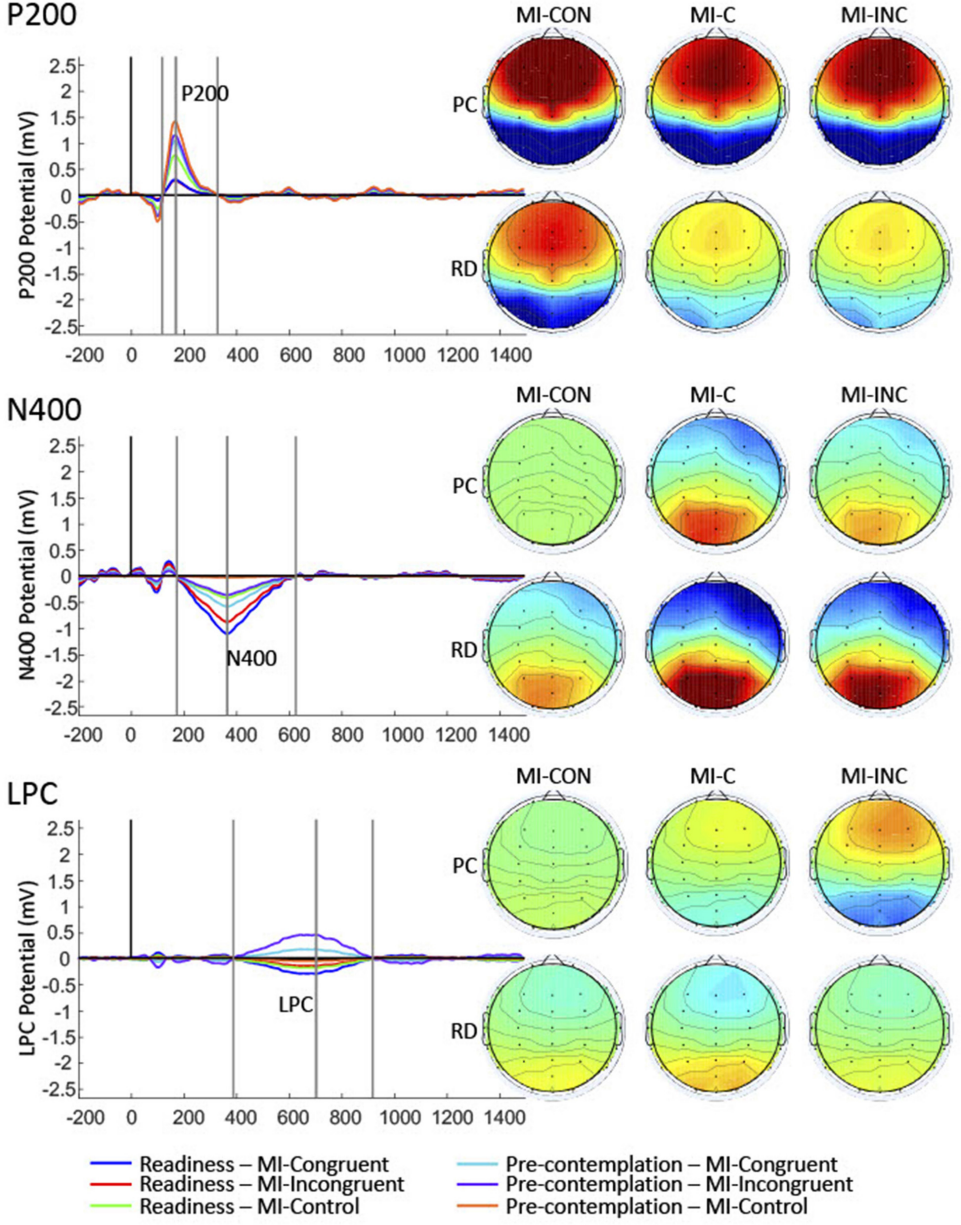
The topography and latency of the P200, N400, and LPC components. The first spatial component of each of the temporal components were then identified. Grand average of the selected temporal-spatial components for each of the conditions were calculated and showed.

The scree plot suggested a 7-factor temporal and 3-factor spatial solution. The components were TF4SF1 (i.e., temporal factor 4, spatial factor 1), TF1SF1, and TF5SF1 (Figures 3, 4). They were identified as the P200, N400, and LPC with the peak latencies and channels as 172 ms at FCz, 368 ms at FP2, and 705 ms at Fz, respectively.

The centro-frontally oriented P200 showed significant Group, [*F*_(1, 30)_ = 4.89, *p* = 0.035] and Condition effects on the amplitudes, [*F*_(2, 60)_ = 15.07, *p* < 0.001], but non-significant interaction effects, [*F*_(2, 60)_ = 0.78, *p* = 0.465]. The RD group had less positive amplitudes than the PC group, *t*(30) = 2.21, *p* = 0.035. The MI-C condition had less positive amplitudes than the MI-CON condition, *t*(60) = 5.03, *p* < 0.001.

The N400 showed significant Condition effect on the amplitudes, [*F*_(2, 60)_ = 8.67, *p* < 0.001]. The Group, [*F*_(1, 30)_ = 1.59, *p* = 0.217] and interaction effects were not significant, [*F*_(2, 60)_ = 0.12, *p* = 0.889]. The MI-C condition showed significantly more negative amplitudes than the MI-CON, *t*(60) = 4.11, *p* < 0.001.

**Figure 5:**
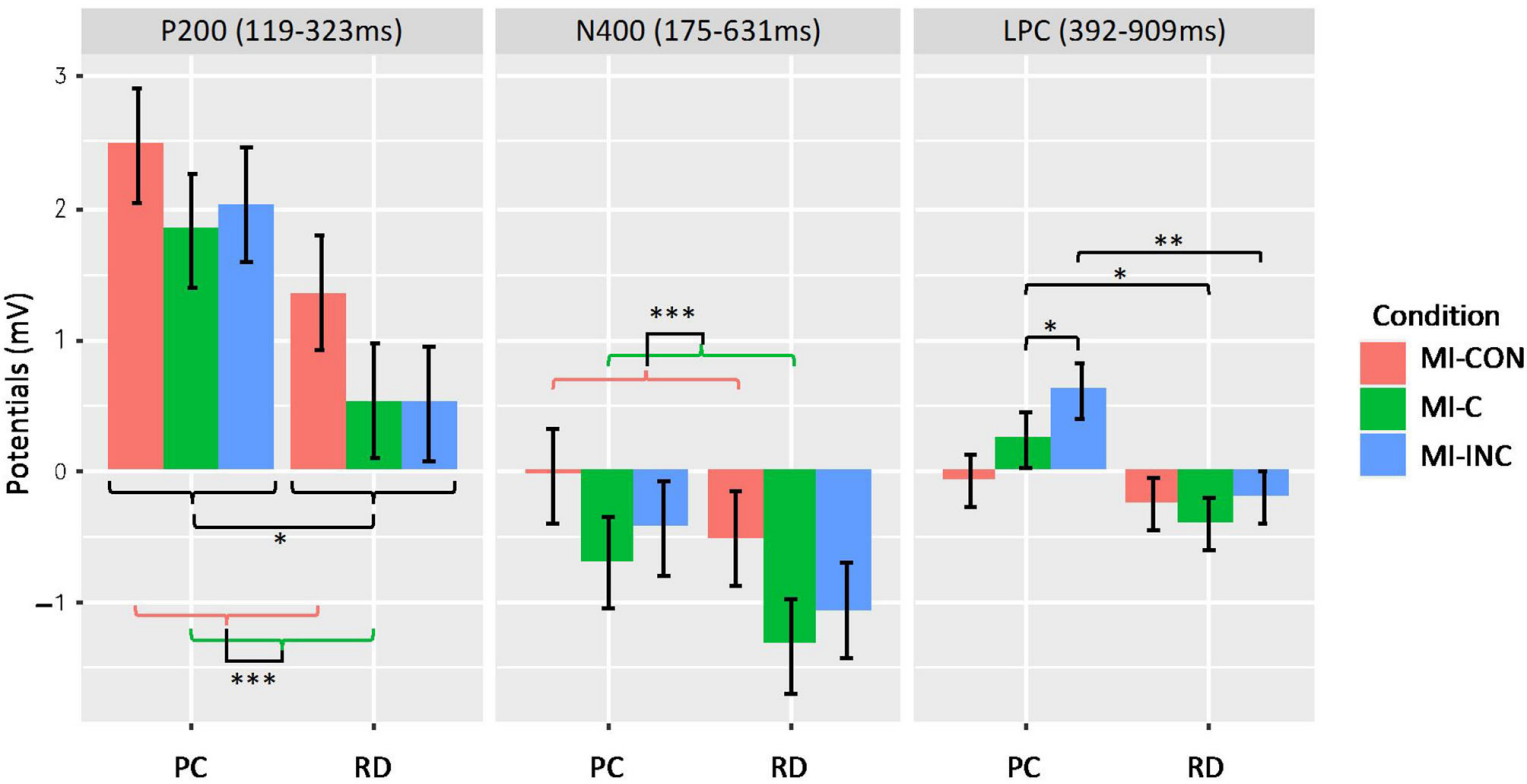
Between-condition contrasts of amplitudes for P200, N400, and LPC for the pre-contemplation (PC) and readiness stage of change group (RD). There was significant main effect of group and condition for P200 potentials, and *post-hoc* test indicated there was significant differences between Ml-CON and Ml-C. There was significant main effect of condition for N400 potentials, and *post-hoc* test showed that there was significant differences between Ml-CON and Ml-C. There was significant group-condition interaction effect for LPC. For PC, Ml-INC was more positive than Ml-C. Between PC and RD, Ml-C and Ml-INC were more positive in PC than that of RD. *p < 0.05, **p < 0.01, ***p < 0.001.

The frontal LPC showed significant Group, [*F*_(1, 30)_ = 4.93, *p* = 0.034], and Condition effects on the amplitudes, [*F*_(2, 60)_ = 4.31, *p* = 0.018]; the Group × Condition effect was also significant, [*F*_(2, 60)_ = 3.18, *p* = 0.049]. The MI-C and MI-INC conditions of the PC group had significantly more positive amplitudes than the RD group (MI-C: *t*(54.3) = −2.23, *p* = 0.03; MI-INC: *t*(54.3) = −2.82, *p* = 0.007). Within the PC group, the MI-INC showed significantly more positive amplitudes than the MI-C, *t*(60) = −2.01, *p* = 0.049. Within the RD group, all the comparisons of the LPC amplitudes were statistically not significant: MI-C vs. MI-INC, *t*(60) = −1.1, *p* = 0.274; MI-C vs. Control, *t*(60) = −0.85, *p* = 0.397. Between-condition contrasts of amplitudes for P200, N400, and LPC are presented in Figure 5.

The main effect of Gender, and Gender × Condition effects were found non-significant on the amplitudes of the P200 {Gender: [*F*_(1, 30)_ = 1.568, *p* = 0.22], Condition: [*F*_(2, 60)_ = 17.53, *p* < 0.001], Interaction: [*F*_(2, 60)_ = 1.963, *p* = 0.15]}, N400 {Gender: [*F*_(1, 30)_ = 0.122, *p* = 0.73], Condition: [*F*_(2, 60)_ = 8.45, *p* < 0.001], Interaction: [*F*_(2, 60)_ = 0.07, *p* = 0.93]}, and LPC {Gender: [*F*_(1, 30)_ = 2.28, *p* = 0.14], Condition: [*F*_(2, 60)_ = 3.881, *p* = 0.03], Interaction: [*F*_(2, 60)_ = 0.12, *p* = 0.89]}. The goodness of fit tests AIC and BIC appeared to converge, suggesting that adding the Gender effect did not significantly modify the results obtained from the original model (Supplementary Table 1). Hence, the rest of the results and their discussions will base on those obtained from the original models.

The correlations between the participants' amplitudes and the linguistic task's reaction times were all not significant. There were between-group differences in the correlational patterns between the ERP's amplitudes and clinical measures scores. In the PC group, the participants' scores on the negative affect subscale of PANAS moderately correlated with the amplitudes of N400 for both the MI-C statements (*r* = 0.607, *p* = 0.016) and the MI-INC statements (*r* = 0.649, *p* = 0.009), which was not the case for the RD group.

## Discussions

This study employed a linguistic method to understand the neural processes associated with reading MI-related content. The main findings are that the MI-related contents appear to elicit emotional perception, cognitive appraisal, and emotional appraisal processes. More importantly, the emotional appraisal process, unique to this study's task design, suggests a possible conflict between the MI-related content and the participants' internal values. The emotional perception and cognitive appraisal processes are based on the significant between-condition contrasts in the frontally oriented P200 and N400. The emotional appraisal process is evident in the significant interaction effects on the frontal LPC. Among all the behavioral variables, only the negative affect in the pre-contemplation participants and the state-trait anxiety in the readiness participants significantly correlated with the captured neural activities, which are interesting findings.

The results suggest differences in emotional states when participants in the two sample groups appraised the MI-related content. Participants in the pre-contemplation stage appear to differ in their emotional appraisal of the MI-related contents from those in the other readiness stages. The more positive-going LPC suggests that the pre-contemplation group participants would have evaluated the MI-related contents more deviated from their values in return-to-work than the readiness group participants. The observation of the pre-contemplation participants' incongruent values is substantiated by the more positive LPC for the MI consistent than for the MI inconsistent contents. In contrast, the readiness group participants would have had similar subjective values (or ambivalence) in evaluating the MI-related contents as no significant differences in the LPC amplitudes were revealed across all types of statements.

### LPC

The most significant results are in the between-group differences in the frontal LPC, which supported the hypothesis of this study. The peak of the amplitude of the LPC was identified as 705 ms around the centro-frontal area. It is comparable to the frontal P600 reported in a linguistic study by Delogu et al. (2019). The frontal LPC is a marker in other studies on processing the emotional contents of words (Wang et al., 2019) and their evaluation (Yang et al., 2018). A recent review of the semantic P600 indicates its role in syntactic manipulation response to semantic anomalies (Leckey and Federmeier, 2020). The more positive going P600 was found to associate with the reprocessing of syntactic after semantic integration (Delogu et al., 2019). In moral studies, more positive-going LPC was found to relate to the processing of value-inconsistent statements when compared with value-consistent statements (Kunkel et al., 2018). Taken together, the more positive-going LPC would reflect appraisal of the syntactic content of the statements which to the injured workers have a salient emotion component. The pre-contemplation group showed more positive-going LPC than the readiness group. It is noteworthy that the differences in the emotional appraisal processes were observed in both the MI consistent and inconsistent contents among the pre-contemplation participants. This is contrary to the readiness participants who showed no significant between-condition differences in the LPC amplitudes. In the MI theory, this indicates ambivalent values (Leffingwell, 2020) among the readiness participants in relating to the semantic contents of return-to-work. Ambivalence, referring to the stage of change model, has been reported as characterizing the values and feelings of those in the contemplation stage (Aasdahl et al., 2018). This matches the sample composition of the readiness group participants of whom eight were classified under contemplation dominance. The heterogeneity in the readiness group may also explain the inconsistency between the within-group LPC amplitude differences and the participants' statement content relevance ratings. Participants in the readiness group found the MI consistent contents more relevant to them than the MI inconsistent contents, which would have been expected to show differences in the LPC amplitudes between the two types of statement contents. Future studies are to consider comparing the potential differences in the LPC between these two readiness groups.

There are two implications of the findings. First, the content effects of both the MI consistent and inconsistent statements were significant during the emotional appraisal process. Differential effects were observed among participants in different stages of change, with the largest effect among those in a pre-contemplation stage. Second, the lack of difference in the elicitation of the LPC between the MI consistent and inconsistent statements provides further support to the ambivalence of value characterizing those in the contemplation stage.

### P200 and N400

The hypothesis on the between-group differences in the amplitudes of P200 was supported, but that on the amplitudes of the N400 was not. Regarding the P200, the centro-frontal positive component peaked at 172 ms. The results of a linguistic study related the frontal P200 to the increases in attention on the targeted words (Yang et al., 2019); while in moral decision-making study, the more positive-going P260 was found to reflect an early affective reaction to dilemma conflict (Sarlo et al., 2012). In this study, both groups showed more positive-going P200 in response to the MI-CON than MI-C trials. This finding suggests that the MI consistent words would have attracted less attention and lower dilemma conflict than the control words among the participants, regardless of their readiness stages. On the other hand, the more positive-going P200 found in the readiness than pre-contemplation group suggests that the MI consistent words would have attracted more attention and higher dilemma conflict in the former than the latter group. These results are in line with those reported in a study conducted by Biau et al. (2018) that the P200 amplitudes reflected the increased attention in processing the relevant information. Thus, for injured workers in a readiness stage, their engagements in the return to work process would have prepared them to perceive the semantic contents as more consistent than the pre-contemplating counterpart.

In contrast to the P200, there was no significant between-group difference in the amplitudes of the frontally oriented N400. Instead, both groups showed more negative-going N400 in response to the MI-C than MI-CON trials. The topography and latency of the N400 revealed in this study are consistent with those reported in Hundrieser and Stahl (2016). As N400 reflects cognitive evaluation of the stimulus contents, the results suggest that participants, regardless of their readiness stages, would have regarded the MI consistent words as value-inconsistent than the control words. Our interpretations of the results are in line with those reported in other studies. For instance, the frontal N400 was associated with the cognitive evaluation of semantically incongruent words (Yang et al., 2018). The more negative-going N400 is associated with value inconsistent statements and expectation-violation information (Peng et al., 2020). In cognitive evaluation, the participants would have accessed the long-term memory and compared their prior knowledge and experience with the MI consistent words (e.g., Bechtold et al., 2019). It is noteworthy that significant correlations between the N400 amplitudes and the level of negative affect existed only among participants in the pre-contemplation group. As more negative N400 amplitudes relate to incongruent value, it is plausible that participants who found the MI consistent contents more incongruent with their values would have a higher level of negative affect. However, this phenomenon did not seem to apply to those who were in the readiness stages.

It is noteworthy that in both P200 and N400, no significant differences were found between the MI-C and MI-INC conditions, which is counterintuitive. It is plausible that the non-significant results would have been attributed to the relatively low salience of the inconsistent words used in the MI-INC trials. Despite the significant between-condition differences, the means of the return to work relevance ratings (RTW-R) ratings were 3.86 vs. 2.79 out of 5.0 for the MI-C and MI-INC, respectively. The low salient effect may be due to only three words were used to convey the inconsistent content. Future study is recommended to address the impact on stimuli's salience on influencing the results.

The significant findings of the P200 and N400 revealed that early processing of the MI consistent content involved emotional perception and cognitive appraisal. In comparison with the late emotional appraisal, these two early processes seem to be independent of the top-down readiness status of the participants. The salience of the MI consistent contents, in the form of a few words, is also demonstrated in this study, highlighting the usefulness of the MI change talks as a medium in counseling.

There are a few limitations to this study. First, the results did not reveal a significant P300 component that has been commonly reported in other studies. This perhaps was due to the design of the MI-C vs. MI-INC and MI-C vs. MI-CON that may have eliminated the P300 common to all conditions. Second, the sample size of the pre-contemplation and readiness groups was relatively small and may have reduced the power of the analyses conducted. The readiness group was composed of participants who were classified under the contemplation, and preparation and action work readiness, which increased within-group heterogeneity and hence further reduced the power. Readers should therefore be cautious when interpreting the results. Future study would be to use a larger size and more homogeneous samples in replicating the study. Other studies could incorporate the EEG components into testing the outcomes of motivational interviewing as an intervention to facilitate behavioral change. Third, this study did not have a non-injured worker control group. The unique characteristics of the injured worker participants, such as their physical impairments and psychological responses to the injuries, could have confounded the between-group results. Fourth, although no significant effects were found, the possibility of the influences, such as emotional processing (Wager et al., 2003; Weisenbach et al., 2014), cannot be eliminated due to the unequal gender composition between the two groups. Future research can consider increasing the sample sizes and incorporating a similar gender composition and non-injured workers as controls to replicate the study.

## Conclusions

This study aimed to construct the neural processes in counseling through the way participants process therapists' statements, including emotional perception (P200), cognitive appraisal (N400), and semantic integration in late emotional appraisal (LPC). The study used abstracted therapists' statements in motivational intervention interventions by focusing on the skills of reflections and affirmations. The results illustrated that the participants had values congruent in processing the motivational interviewing consistent statements. This may provide evidence to support the efficacy of the semantic contents of MI statements as the technical component in a MI mechanism. The LPC interaction effect confirms the differentiation of participants in various stages of change in counseling, and that they are likely to have different subjective values in processing the therapists' statements.

## Data Availability Statement

The raw data supporting the conclusions of this article will be made available by the authors, without undue reservation.

## Ethics Statement

The studies involving human participants were reviewed and approved by ethics committee of the Department of Rehabilitation Sciences at The Hong Kong Polytechnic University. The patients/participants provided their written informed consent to participate in this study.

## Author Contributions

KH: conceptualization, methodology, software, formal analysis, and writing-original draft preparation. CW: formal analysis, software, data curation, and writing-original draft preparation. AS: supervision and validation. TL: writing-reviewing and editing. CC: conceptualization supervision, visualization, and writing-reviewing and editing. All authors contributed to the article and approved the submitted version. KH and CW contributed equally to this article as first authors.

## Funding

This study is supported by the Peter T. C. Lee Endowed Chair Professorship of The Education University of Hong Kong held by CC.

## Conflict of Interest

The authors declare that the research was conducted in the absence of any commercial or financial relationships that could be construed as a potential conflict of interest.

## Publisher's Note

All claims expressed in this article are solely those of the authors and do not necessarily represent those of their affiliated organizations, or those of the publisher, the editors and the reviewers. Any product that may be evaluated in this article, or claim that may be made by its manufacturer, is not guaranteed or endorsed by the publisher.
