# Supplementary material for: Cognitive and Emotional Appraisal of Motivational Interviewing Statements: An Event-Related Potential Study
Source: Front Hum Neurosci. 2021 Sep 22;15:727175. doi: 10.3389/fnhum.2021.727175 (PMC8494474; doi:10.3389/fnhum.2021.727175)
Supplement: Supplementary file 1 [file Data_Sheet_1.docx]

**Detailed Descriptions of the Experimental Task**

**Construction MI Statements.** There were three types of statements: MI-congruent, MI-incongruent, and a control. The statement stimuli were made up of: 1) seven Chinese characters; 2) the same syntactic structure; 3) the last three characters critical to the behavioral responses (three-critical-characters; Li et al., 2016). The contents of the MI-congruent statements were extracted from the transcripts of the first author, who is a member of the Motivational Interviewing Network of Trainers (MINT), of one-hour MI intervention sessions with five injured workers (Miller & Rollnick, 2013). A total of 120 short MI-congruent statements (MI-C) were composed, conveying positive values consistent with the MI approach. Another 120 MI-incongruent statements (MI-INC) were composed, sharing a similar structure and format to the corresponding MI-C statements but with negative values unsupportive of return-to-work. The 120 control statements (MI-CON) had the three-critical-characters of fruit names, considered to be contextually neutral (Chen et al., 2013). Examples of the MI-C and MI-INC statements were “return to work is important” (重返工作好重要) and “return to work is not easy” (重返工作不容易), respectively. An example of MI-CON is “There is four fuji apple” (富士蘋果有肆個). An additional feature specific to Chinese characters is that the mean number of strokes of the critical characters for the three types of statements were comparable, ranging from 28.3 to 28.5.

**The Task.** The delivery of the statements and the type of response adopted the rapid serial visual presentation (RSVP) method commonly used in psycholinguistic studies (Chen et al., 2013; Hundrieser & Stahl, 2016). The design of the statement trial is presented in Figure 1. The participants read a MI-C statement presented on the computer screen in the form of three consecutive texts and then gave a response to the question: “Would it enhance your motivation for return to work?” (能增加你的重返工作動機嗎?) by pressing on one of the five keys on the keyboard (1 = “No, it very much would not.” (完全不能); to 5 = “Yes, it very much would.” (完全可以). The same question was repeated in all the MI-C and MI-INC statements. The responses to the MI-CON statements indicated the number of fruits conveyed in the text, such as a “4” response to the “There is four fuji apples” (富士蘋果有肆個). After completing the task, the participants rated the statements according to their relevance to their return-to-work situation, using a 5-point rating scale (1 = “very irrelevant” (沒有太大的關切) to ; 5 = “very relevant” (有緊密的關切).

Supplementary Table 1. Model comparison for the effect of gender on linear-mixed models of P200, N400 and LPC potentials

| **Models** | **Df** | **AIC** | **BIC** |
| --- | --- | --- | --- |
| ***P200*** |  |  |  |
| Model 1: Potential ~ Group * Condition + (1\|Subject) | 8 | 265.7 | 285.7 |
| Model 2: Potential ~ Gender * Condition + (1\|Subject) | 8 | 266.4 | 286.4 |
| Model 3: Potential ~ Group * Condition + Gender + (1\|Subject) | 9 | 266.9 | 289.4 |
| Model 4: Potential ~ Group * Condition * Gender + (1\|Subject) | 14 | 267.5 | 302.5 |
|  |  |  |  |
| ***N400*** |  |  |  |
| Model 1: Potential ~ Group * Condition + (1\|Subject) | 8 | 274.7 | 294.7 |
| Model 2: Potential ~ Gender * Condition + (1\|Subject) | 8 | 276.2 | 296.2 |
| Model 3: Potential ~ Group * Condition + Gender + (1\|Subject) | 9 | 276.6 | 299.1 |
| Model 4: Potential ~ Group * Condition * Gender + (1\|Subject) | 14 | 283.8 | 318.8 |
|  |  |  |  |
| ***LPC*** |  |  |  |
| Model 1: Potential ~ Group * Condition + (1\|Subject) | 8 | 198.0 | 218.0 |
| Model 2: Potential ~ Gender * Condition + (1\|Subject) | 8 | 206.2 | 226.2 |
| Model 3: Potential ~ Group * Condition + Gender + (1\|Subject) | 9 | 198.7 | 221.2 |
| Model 4: Potential ~ Group * Condition * Gender + (1\|Subject) | 14 | 208.4 | 243.4 |

Note: Model 1 was the original model. Model 2 tested the Gender effect without Group variable. Model 3 assumed simple effect of Gender. Model 4 assumed a full interaction effects. Goodness of fit tests (both AIC and BIC values) indicated that models without the effect of gender were more proper for P200, N400 and LPC components.
